# A Novel Germline *SDHA* Gene Mutation and Co-Occurring Somatic *KIT* Activating Mutation in a Patient With Pediatric Central Nervous System Germ Cell Tumor: Case Report

**DOI:** 10.3389/fonc.2022.835220

**Published:** 2022-05-16

**Authors:** Xizan Yue, Bo Liu, Tiantian Han, Ningning Luo, Guanghua Lu, Didi Guo, Fanfeng Bu, Guangyu Wang

**Affiliations:** ^1^ Department of Neurosurgery, Qilu Children’s Hospital of Shandong University, Jinan, China; ^2^ The Medical Department, Jiangsu Simcere Diagnostics Co., Ltd., Nanjing, China; ^3^ The Medical Department, Nanjing Simcere Medical Laboratory Science Co., Ltd., Nanjing, China; ^4^ The State Key Lab of Translational Medicine and Innovative Drug Development, Jiangsu Simcere Diagnostics Co., Ltd., Nanjing, China

**Keywords:** central nervous system germ cell tumors, *SDHA* gene, *KIT* gene, pediatric, germline

## Abstract

Central nervous system germ cell tumors (CNS GCTs) are a heterogeneous group of primary CNS tumors. GCTs are more common and mostly observed in pediatric and young adult patients. CNS GCTs are divided into germinomas and non-germinomatous germ cell tumors (NGGCTs), with different therapeutic strategies depending on diagnosis. Herein, we report a patient with pediatric central nervous system germinoma harboring a somatic *KIT* p.Y823D and a heterozygous germline *SDHA* p. T396Nfs*14 mutation detected by next generation sequencing. After surgery, the patient received chemotherapy (temozolomide + nedaplatin + etoposide). This is the first report of a Chinese pediatric patient with CNS GCT harboring concurrent germline *SDHA* and somatic *KIT* mutation, which enriches molecular profiles of CNS GCTs and provides more molecular evidence of clinical diagnosis and potential targeted therapy in CNS GCTs.

## Introduction

Central nervous system (CNS) germ cell tumors (GCTs) are rare tumors originating primarily from midline locations, including pineal and suprasellar regions. The 2021 world health organization (WHO) classification of tumors of the CNS identified 8 GCTs subtypes: mixed germ cell tumor; germinoma (GE); nongerminomas germ cell tumors (NGGCTs) including choriocarcinoma, yolk sac tumor, embryonal carcinoma; and teratoma classified further as mature teratoma, immature teratoma and teratoma with somatic-type malignancy (1). The incidence of CNS GCTs was once considered more prevalent in East Asia than in the Western Countries (2). But the study by McCarthy et al. revealed that the incidence of primary GCTs between Japan and the United States is similar, and the gender-based patterns by location and high rates of survival are similar too (3). The median age at which CNS GCTs are diagnosed is 10–14 years, and there is a significant predominance in males (4, 5). GE has a relatively better prognosis than NGGCTs, with a 5-year survival rate of approximately 90%, which in NGGCTs is 75–82% (5, 6). CNS GCTs are very responsive to radiation therapy/chemotherapy. NGGCTs usually secrete elevated alpha-fetoprotein (AFP) and/or beta-human chorionic gonadotropin (beta-hCG) into serum and cerebrospinal fluid, so AFP and beta-hCG are helpful in distinguishing GEs and NGGCTs. Different tests including CT or MRI scans, tumor markers in serum and cerebrospinal fluid, and histopathology are used to diagnose GCT, but there are still difficulties (7, 8). To date, there are limited large-scale data of GCTs in China, with only 2 large studies (9, 10).

Molecular characterization studies have shown that *KIT* or *KRAS* gene mutations are present in about 25% of seminomas, yet it is rare in non-seminomatous germ cell tumor (11). Gain of chromosome 12p exists in almost all testicular GCTs (12). The gain of chromosome 12p is observed in CNS GCTs with lower frequency (13). With the application of next-generation sequencing (NGS) analysis in recent years, the combination of activated KIT signaling pathway and complex chromosomal anomalies, especially gain of chromosome 12p seems to be the key molecular drive of gonadal GCTs pathogenesis (11, 12). Recently, whole-exome sequencing of CNS GCTs reveals mutations in the KIT-RAS-MAPK or AKT-MTOR pathways, including *KIT* (26%), *KRAS/NRAS* (20%), *CBL* (11%), *MTOR* (8%), and *NF1* (3%) (14). Moreover, a rare germline variant of *JMJD1C* S880P is associated with CNS GCTs, especially in Japanese. Germline *SDHA* mutation has not been reported in GCTs (14). Nonetheless, the complete molecular characterization of CNS GCTs in Chinese has not been achieved. In the present case, for the first time, we report a Chinese CNS germinoma patient with a *KIT* hotspot somatic mutation concurrent with a novel *SDHA* germline mutation that showed distinct early-onset.

## Case Presentation

**Figure 1:**
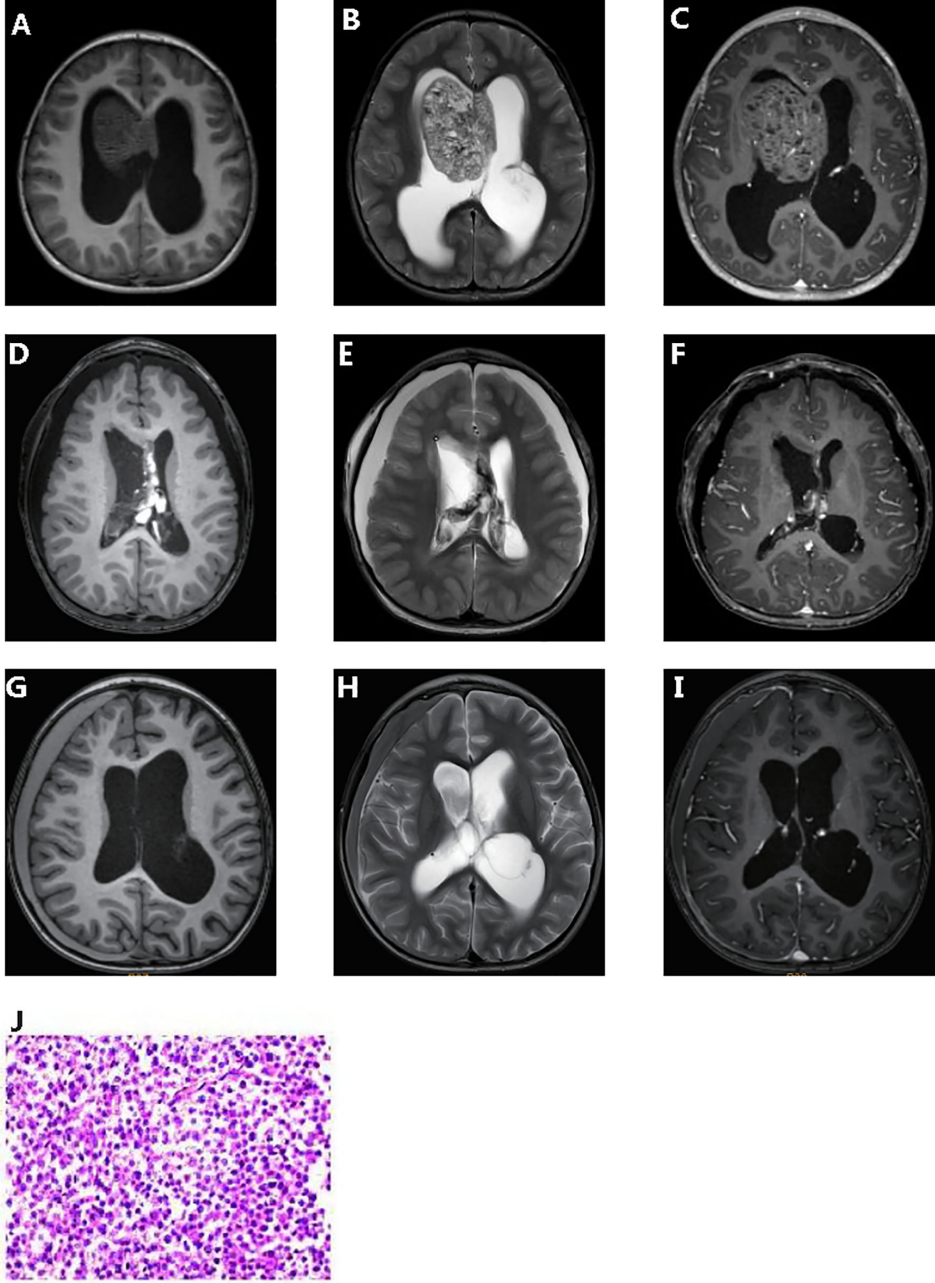
Clinicopathologic features of patient. Pre-operative T1-weighted **(A)** and T2- weighted **(B)** dynamic contrast enhanced MRI **(C)**. Post-operative T1-weighted **(D)** and T2- weighted **(E)** dynamic contrast enhanced MRI **(F)**. 5 months post-operative T1-weighted **(G)** and T2- weighted **(H)** dynamic contrast enhanced MRI **(I)**. Hematoxylin and eosin **(J)**.

A 4-year-old male toddler presented to our hospital due to the elevated serum HCG levels, produced by the tumor. On examination, the muscle strength of the lower limbs was grade 4 and the skin of the whole body was hairy, with a beard and pubic hair. The development of external genitalia was significantly larger than the boys of the same age. An irregular cafe-au-lait macule could be seen on the right forehead, which might be considered as a sign of the syndrome. Brain magnetic resonance imaging (MRI) scans revealed a space-occupying lesion in the right lateral ventricle with hydrocephalus (**Figures 1A**–**C**). Increasing levels of β-hCG (207.72 U/L—NV 0-5) were found in serum. The patient had epiphyseal dysplasia of the lower limbs with a slight limp in the right lower limb. The patient underwent space-occupying resection of the lesions and external ventricular drain (**Figures 1D**–**F**). Microscopical analysis indicated that the tumor was a malignant tumor of germ cell origin without any nongerminomas germ cell tumors components (**Figure 1J**). Immunochemistry was performed and showed AFP/HCG negative and PLAP/CD117 positive by postoperative tissue, and probably the HCG-producing trophoblastic giant cells have been missed. The Ki67 proliferation index of hot spots was high (50%-60%). After surgery, acne improved and β-hCG returned to normal levels. Combined with histological morphology and immunohistochemistry, the final diagnosis was a secreting CNS germinoma.

**Figure 2:**
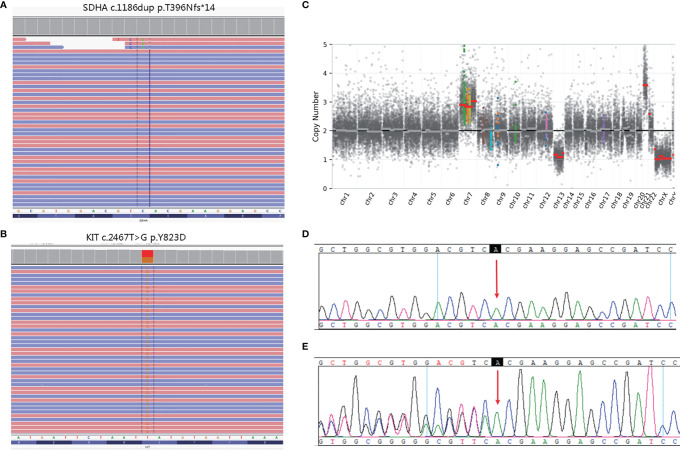
Molecular pathology data of patient. Next generation sequencing (NGS) revealing a germline *SDHA* c.1186dup variant **(A)**, and a somatic *KIT* c.2467T>G variant **(B)**. Copy number variation (CNV) in the whole genome **(C)**. The unaffected father of the patient carried the heterozygous *SDHA* c.1186dup mutation **(E)**, whereas the healthy mother did not **(D)**.

The white blood cell and tumor tissue specimens of the patient were collected for NGS detection based on a panel including 539 cancer-related genes (Simceredx). By comprehensive genomic profiling, somatic *KIT* p.Y823D (AF 46.29%) and *SDHA* p. T396Nfs*14 (AF 48.35%) was detected but no second hits or loss of heterozygosity (LOH) in tumor tissue (**Figures 2A, B**). Copy number variations (CNV) including the gain of chromosome 7 and 21, and loss of chromosome 13 were identified (**Figure 2C**). Due to the younger age of diagnosis with a cafe-au-lait macule phenotype, we confirmed the germline mutation in leukocytes. No cafe-au-lait macule-associated tumor syndrome mutation was identified, but *SDHA* p. T396Nfs*14 was confirmed a heterozygous germline mutation. Further analysis by Sanger sequencing identified that the *SDHA* heterozygous mutation originated from his father with a normal phenotype (**Figures 2D, E**). The patient didn’t have familial cancer history.

The patient received chemotherapy (temozolomide + nedaplatin + etoposide) and radiation therapy after surgery. Computed tomography scan after chemotherapy in of the brain showed a few effusions in the right subdural. Up to the article submission, there was no tumor recurrence (**Figures 1G**–**I**).

## Discussion and Conclusion

In our case, we found concurrent germline *SDHA* and somatic *KIT* mutations, which was not reported in CNS GCTs. *SDHA* gene encodes a major subunit of Succinate dehydrogenase, a complex of the mitochondrial respiratory chain whose subunits are encoded by *SDHA*, *SDHB*, *SDHC*, *SDHD*, *SDHAF1*, and *SDHAF2*. As tumor suppressor genes, aberrations in the SDH complex genes result in the SDH complex deficiency (SCD) syndrome, an autosomal dominant disease with the occurrence of multiple tumors including gastrointestinal stromal tumors (GISTs), paragangliomas (PGLs), pheochromocytomas (PCCs), renal cell carcinomas (RCCs), and others (15). According to the paper by Korpershoek et al., the penetrance of *SDHA* - associated apparently sporadic paragangliomas and pheochromocytoma is low (16). Recently, germline mutations in SDHx were also reported in germ cell tumors including *SDHB* p.L157W in mediastinal germ cell tumor and *SDHD* p.W43* in testicular seminoma (17, 18). The patient has no evidence of PGLs/PCCs and his father carries the same *SDHA* mutation without tumor phenotype, which may be related to the incomplete penetrance of SCD syndrome.

The p. T396Nfs*14 (c.1186dup) mutation is located in exon 9 of the *SDHA* gene. The variant is predicted to result in a premature stop codon at position 409 of protein likely causing an absence or loss of C-terminal domain protein product (19). According to the guideline of the American College of Medical Genetics and Genomics (ACMG), we defined the *SDHA* mutation as a pathogenic variant. *KIT* p.Y823D mutation lies in the protein kinase domain of the Kit protein conferring a gain of function on Kit and then activates the KIT-RAS-MAPK pathway (20). Although the current evidence is insufficient to establish a clinical relationship for GCTs and *SDHx* mutations. Considering the early onset of the disease in this patient, we speculate that *SDHA* germline mutation promotes a more aggressive behavior of GCTs related to the KIT-RAS-MAPK pathway potentially, by activating the pseudo-hypoxic pathway to increased ROS, angiogenesis, and cell proliferation in SDH-deficient tumors (21).

At present, surgery, radiotherapy, and chemotherapy can all improve the outcomes of CNS GCTs. The genomic analysis could provide the application of TKIs in in CNS GCTs. In CNS GCTs, *KIT* mutations were mainly concentrated in exon 17, followed by exon 11. *KIT* p.Y823D in GCT patients was reported to be sensitive to sorafenib and resistant to imatinib and Sunitinib (14). Tyrosine kinase inhibitors (TKIs) targeting KIT have been approved in other tumors. *KIT* mutations and *SDHx* germline mutations are also common in gastrointestinal stromal tumors, and distinct from our case, they are usually mutually exclusive (22). The possible synergistic or antagonistic effects of the co-mutation on the drug are unknown.

In conclusion, we for the first time report a 4-year-old Chinese male child with CNS GCT harboring concurrent germline *SDHA* and somatic *KIT* mutation, which showed the necessity to detect germline mutations in CNS GCTs, especially in children with CNS GCTs. In addition, the relationship between *SDHA* germline mutations and the clinical diagnosis, prognosis, and therapy of CNS GCTs needs to be further investigated.

## Data Availability Statement

The original contributions presented in the study are included in the article. Further inquiries can be directed to the corresponding author.

## Ethics Statement

Written informed consent was obtained from the patient for the publication of any potentially identifiable images or data included in this article.

## Author Contributions

GW designed the study. XY, BL drafted the manuscript. TH participated in the manuscript. TH, NL analyzed the literature. NL, GL revised the manuscript. DG, FB participated in the revision. All authors contributed to the article and approved the submitted version.

## Conflict of Interest

Authors NL, TH, DG, GL, and FB are employed by the company Jiangsu Simcere Diagnostics Co., Ltd.

The remaining authors declare that the research was conducted in the absence of any commercial or financial relationships that could be construed as a potential conflict of interest.

## Publisher’s Note

All claims expressed in this article are solely those of the authors and do not necessarily represent those of their affiliated organizations, or those of the publisher, the editors and the reviewers. Any product that may be evaluated in this article, or claim that may be made by its manufacturer, is not guaranteed or endorsed by the publisher.

## References

[1] Louis DN Perry A Wesseling P Brat DJ Cree IA Figarella-Branger D. The 2021 WHO Classification of Tumors of the Central Nervous System: A Summary. Neuro Oncol (2021) 23(8):1231–51. doi: 10.1093/neuonc/noab106 PMC8328013 34185076

[2] Packer RJ Cohen BH Cooney K. Intracranial Germ Cell Tumors. Oncologist (2000) 5:312–20. doi: 10.1634/theoncologist.2000-0312 10964999

[3] McCarthy BJ Shibui S Kayama T Miyaoka E Narita Y Murakami M. Primary CNS Germ Cell Tumors in Japan and the United States: An Analysis of 4 Tumor Registries. Neuro Oncol (2012) 14:1194–200. doi: 10.1093/neuonc/nos155 PMC3424216 22869621

[4] Shibui S. Report of Brain Tumor Registry of Japan (2001–2004). Neurol Medico-Chirurgica (2014) 54(suppl.1):9–102. doi: 10.2176/nmc.sup.2014-0001

[5] Gittleman H Cioffi G Vecchione-Koval T Ostrom QT Kruchko C Osorio DS. Descriptive Epidemiology of Germ Cell Tumors of the Central Nervous System Diagnosed in the United States From 2006 to 2015. J Neuro-Oncol (2019) 143(2):251–60. doi: 10.1007/s11060-019-03173-4 31025275

[6] Calaminus G Frappaz D Kortmann RD Krefeld B Saran F Pietsch T. Outcome of Patients With Intracranial Non-Germinomatous Germ Cell Tumors-Lessons From the SIOP-CNS-GCT-96 Trial. Neuro Oncol (2017) 19(12):1661–72. doi: 10.1093/neuonc/nox122 PMC5716085 29048505

[7] Takami H Fukuoka K Fukushima S Nakamura T Mukasa A Saito N. Integrated Clinical, Histopathological, and Molecular Data Analysis of 190 Central Nervous System Germ Cell Tumors From the iGCT Consortium. Neuro-Oncology (2019) 21:12: 1565–77. doi: 10.1093/neuonc/noz139 PMC6917411 31420671

[8] Murray MJ Bartels U Nishikawa R Fangusaro J Matsutani M Nicholson JC. Consensus on the Management of Intracranial Germ-Cell Tumours. Lancet Oncol (2015) 16:9: e470–7. doi: 10.1016/S1470-2045(15)00244-2 26370356

[9] Fang LX Zhu MH Xu SX Li ZY Qi S. Diagnostic Analysis of 162 Casesof Primary Intracranial Germ Cell Tumors (in Chinese). Chin J Neurosurg (2014) 30:541–4. doi: 10.3760/cma.j.issn.1001-2346.2014.06.001

[10] Zhou ZG Yan HL Chen BG Hu ZP Deng LT Zhou XH. 125cases of Diagnostic Evaluation of Primary Intracranial Germ Cell Tumors (in Chinese). J Clin Surg (2017) 25:658–60. doi: 10.3969/j.issn.1005-6483.2017.09.006

[11] Sheikine Y Genega E Melamed J Lee P Reuter VE Ye H. Molecular Genetics of Testicular Germ Cell Tumors. Am J Cancer Res (2012) 2(2):153–67. PMC3304567 22432056

[12] Reuter VE. Origins and Molecular Biology of Testicular Germ Cell Tumors. Mod Pathol (2005) 18 Suppl 2:S51–60. doi: 10.1038/modpathol.3800309 15761466

[13] Sukov WR Cheville JC Giannini C Carlson AW Shearer BM Sinnwell JP. Isochromosome 12p and Polysomy 12 in Primary Central Nervous System Germ Cell Tumors: Frequency and Association With Clinicopathologic Features. Hum Pathol (2010) 41(2):232–8. doi: 10.1016/j.humpath.2009.07.017 19801160

[14] Wang L Yamaguchi S Burstein MD Terashima K Chang K Ng HK. Novel Somatic and Germline Mutations in Intracranial Germ Cell Tumours. Nature (2014) 511(7508):241–5. doi: 10.1038/nature13296 PMC4532372 24896186

[15] Hensen EF Bayley JP. Recent Advances in the Genetics of SDH-Related Paraganglioma and Pheochromocytoma. Familial Cancer (2011) 10(2):355–63. doi: 10.1007/s10689-010-9402-1 PMC3100491 21082267

[16] Korpershoek E Favier J Gaal J Burnichon N van Gessel B Oudijk L. SDHA Immunohistochemistry Detects Germline SDHA Gene Mutations in Apparently Sporadic Paragangliomas and Pheochromocytomas. J Clin Endocrinol Metab (2011) 96(9):E1472–6. doi: 10.1210/jc.2011-1043 21752896

[17] De Filpo G Cilotti A Rolli L Pastorino U Sonzogni A Pradella S. SDHx and Non-Chromaffin Tumors: A Mediastinal Germ Cell Tumor Occurring in a Young Man With Germline SDHB Mutation. Medicina (2020) 56(11):561. doi: 10.3390/medicina56110561 PMC7693473 33113876

[18] Galera-Ruiz H Gonzalez-Campora R Rey-Barrera M Rollón-Mayordomo A Garcia-Escudero A Fernández-Santos JM. W43X SDHD Mutation in Sporadic Head and Neck Paraganglioma. Anal Quant Cytol Histol (2008) 30(2):119–23. 18561749

[19] Renkema GH Wortmann SB Smeets RJ Venselaar H Antoine M Visser G. SDHA Mutations Causing a Multisystem Mitochondrial Disease: Novel Mutations and Genetic Overlap With Hereditary Tumors. Eur J Hum Genet (2015) 23(2):202–9. doi: 10.1038/ejhg.2014.80 PMC4297908 24781757

[20] Caenepeel S Renshaw-Gegg L Baher A Bush TL Baron W Juan T. Motesanib Inhibits Kit Mutations Associated With Gastrointestinal Stromal Tumors. J Exp Clin Cancer Res (2010) 29(1):1–8. doi: 10.1186/1756-9966-29-96 20633291 PMC2912835

[21] Vicha A Taieb D Pacak K. Current Views on Cell Metabolism in SDHx-Related Pheochromocytoma and Paraganglioma. Endocrine-Related Cancer (2014) 21(3):R261. doi: 10.1530/ERC-13-0398 24500761 PMC4016161

[22] Corless CL Barnett CM Heinrich MC. Gastrointestinal Stromal Tumours: Origin and Molecular Oncology. Nat Rev Cancer (2011) 11(12):865–78. doi: 10.1038/nrc3143 22089421

